# Deep Learning-Based Image Reconstruction for CT Angiography of the Aorta

**DOI:** 10.3390/diagnostics11112037

**Published:** 2021-11-03

**Authors:** Andra Heinrich, Felix Streckenbach, Ebba Beller, Justus Groß, Marc-André Weber, Felix G. Meinel

**Affiliations:** 1 Institute of Diagnostic and Interventional Radiology, Pediatric Radiology and Neuroradiology, University Medical Center Rostock, 18057 Rostock, Germany; andra.heinrich@med.uni-rostock.de (A.H.); felix.streckenbach@med.uni-rostock.de (F.S.); Ebba.Beller@med.uni-rostock.de (E.B.); Marc-Andre.Weber@med.uni-rostock.de (M.-A.W.); 2 Center for Transdisciplinary Neurosciences Rostock, University Medical Center Rostock, 18057 Rostock, Germany; 3 Division of Vascular Surgery, Department of Surgery, University Medical Center Rostock, 18057 Rostock, Germany; Justus.Gross@med.uni-rostock.de

**Keywords:** deep learning, image processing, angiography, computed tomography, aorta

## Abstract

To evaluate the impact of a novel, deep-learning-based image reconstruction (DLIR) algorithm on image quality in CT angiography of the aorta, we retrospectively analyzed 51 consecutive patients who underwent ECG-gated chest CT angiography and non-gated acquisition for the abdomen on a 256-dectector-row CT. Images were reconstructed with adaptive statistical iterative reconstruction (ASIR-V) and DLIR. Intravascular image noise, the signal-to-noise ratio (SNR) and the contrast-to-noise ratio (CNR) were quantified for the ascending aorta, the descending thoracic aorta, the abdominal aorta and the iliac arteries. Two readers scored subjective image quality on a five-point scale. Compared to ASIR-V, DLIR reduced the median image noise by 51–54% for the ascending aorta and the descending thoracic aorta. Correspondingly, median CNR roughly doubled for the ascending aorta and descending thoracic aorta. There was a 38% reduction in image noise for the abdominal aorta and the iliac arteries, with a corresponding improvement in CNR. Median subjective image quality improved from good to excellent at all anatomical levels. In CT angiography of the aorta, DLIR substantially improved objective and subjective image quality beyond what can be achieved by state-of-the-art iterative reconstruction. This can pave the way for further radiation or contrast dose reductions.

## 1. Introduction

CT angiography is the predominant imaging modality for diagnosis, treatment planning and follow-up of aortic pathologies [1]. With modern CT scanners, CT angiography offers excellent spatial and temporal resolution, short examination times and is readily available both in the emergency setting in patients with acute aortic syndrome and for elective surveillance, pre- or post-treatment imaging.

Despite the marked reduction of radiation exposure with state-of-the-art techniques such as reducing the tube voltage, prospective ECG triggering and tube current modulation, cumulative radiation exposure is still a concern in those patients requiring repeated follow-up examinations [2]. With reductions in radiation dose, higher levels of image noise can complicate accurate assessment [3].

Iterative image reconstruction has been developed to decrease image noise and thus preserve or even improve image quality at a reduced dose [4]. Since 2008, all major CT vendors have developed various generations of iterative reconstruction algorithms, and iterative reconstruction has thus replaced filtered back projection as the state-of-the-art method in CT image reconstruction [5]. More complex iterative reconstruction algorithms implement models of the acquisition process, image statistics and system geometry in the reconstruction process; this approach has been referred to as model-based iterative reconstruction (MBIR) [5]. However, MBIR images can be notably degraded due to low-frequency noise and usually require higher processing power and longer processing times [6]. 

Algorithms that combine both analytical and iterative methods have been referred to as hybrid iterative reconstruction algorithms [5]. A more recent version of a manufacturer-specific hybrid iterative reconstruction is adaptive statistical iterative reconstruction V (ASIR-V), with a less complex model than MBIR. The aim is to optimize noise, object and physics modelling with more aggressive noise reduction, enable much faster image reconstruction and improve perceived image quality [7,8,9]. 

The most recent development in the field of CT image reconstruction is the development of deep-learning-based image reconstruction (DLIR), which uses deep convolutional models based on neural networks [10]. This technique contains a filter for noise and artefact reduction, promises high resolution and allows the detection of low-contrast lesions [11]. Highly selected, essentially artifact-free image datasets of both phantoms and patients are used in training the deep-learning-based algorithms [12]. 

The purpose of this study was to evaluate the impact of a novel, commercial DLIR algorithm on objective and subjective image quality in CT angiography of the aorta using a state-of-the-art, advanced, iterative reconstruction algorithm as the reference standard.

## 2. Materials and Methods

### 2.1. Patient Selection and Study Design

This study was conducted as a retrospective single-center study. The study protocol was approved by the responsible institutional review board (blinded) with a waiver of informed consent and performed in accordance with the Declaration of Helsinki. This study included 51 consecutive adult patients who had been referred to our department for a clinically indicated aortic CT angiography between May and August 2020 and were identified through a retrospective search of our radiology information system (Centricity 5.0, GE Healthcare). We excluded repeat examinations of identical patients, CT examinations that were not reconstructed with DLIR and examinations performed without ECG-gating for the thoracic aorta.

### 2.2. CT Acquisition Protocol

CT acquisition parameters are summarized in Table 1. The patients were examined on a 256-detector-row CT (Revolution CT, GE Healthcare, Chicago, IL, USA). We used a prospectively ECG-triggered axial scan of the thoracic aorta (in two sequential steps each covering up to 160 mm) immediately followed by a non-ECG-gated helical acquisition of the abdominal aorta. In eight patients, only the thoracic aorta was imaged as clinically indicated. *Z*-axis coverage was from the lung apex to just below the diaphragm in cases of the thoracic aorta. If the entire aorta was to be scanned, the end of the scan range was placed at the level of the femoral head. Gantry rotation time was 0.28 s, detector coverage was 160 mm (thoracic) and 80 mm (abdominal), tube voltage was 100 kV and attenuation-based tube current modulation was used with a reference noise index of 25. Nineteen of the fifty-one patients were in atrial fibrillation during the scan. All patients were asked to hold their breath in inspiration during the examination. Eighty milliliters of an intravenous contrast agent (Imeron^®^ 400 mg/mL, Bracco Imaging, Milan, Italy) was injected at a flow rate of 4 mL/s, followed by 40 mL of saline injected at the same flow rate. A bolus triggering algorithm was used, which automatically started the scan 3 s after a prespecified threshold of 150 HU was reached in the descending thoracic aorta. For the purpose of this study, we analyzed only the arterial-phase images. In some patients, additional phases were performed, such as a non-contrast scan in acute aortic syndrome or a delayed phase in patients after endovascular repair. These additional phases were not evaluated in our study. 

### 2.3. CT Image Reconstruction

For all patients, images were reconstructed on the Revolution CT scanner (software version 2.1B) with both hybrid iterative reconstruction (ASIR-V at 60% strength) and deep-learning-based reconstruction (“TrueFidelity^TM^”, GE Healthcare, Chicago, IL, USA–setting: high strength). We chose to use ASIR-V 60% since this was the default setting for CT angiography as provided by the manufacturer. The slice thickness was 0.625 mm with a 0.625 mm increment. The high-definition standard (HD Stnd) reconstruction kernel was used for all ASIR-V reconstructions. For DLIR, the high-definition standard (HD Stnd) kernel was used for the ECG-triggered chest images and the standard (Stnd) kernel was used for the non-ECG-gated images of the abdominal aorta. The reconstruction time was 25 frames per second for ASIR-V and approximately 10 frames per second for DLIR. Thus, the typical reconstruction time for a thin-slice reconstruction of the entire aorta (approximately 1000 images at 0.625 mm slice thickness) was 40 s for ASIR-V and 100 s for DLIR.

**Figure 1:**
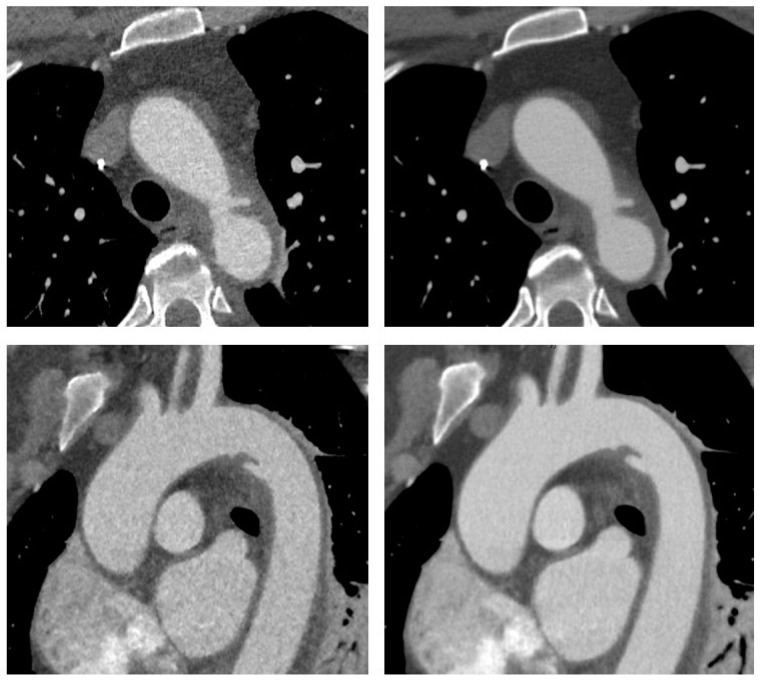
CT angiography of the aorta in a 54-year old woman involved in a motor vehicle accident. Transverse (**upper row**) and sagittal oblique (**lower row**) reconstructions are shown with adaptive statistical iterative reconstruction V (ASIR-V 60%, **left**) and deep learning-based image reconstruction–high strength (DLIR-H, **right**). Images demonstrate traumatic injury to the aortic isthmus with adjacent hematoma indicating contained aortic rupture. The patient was hemodynamically stable and was successfully treated with implantation of an aortic stentgraft.

**Figure 2:**
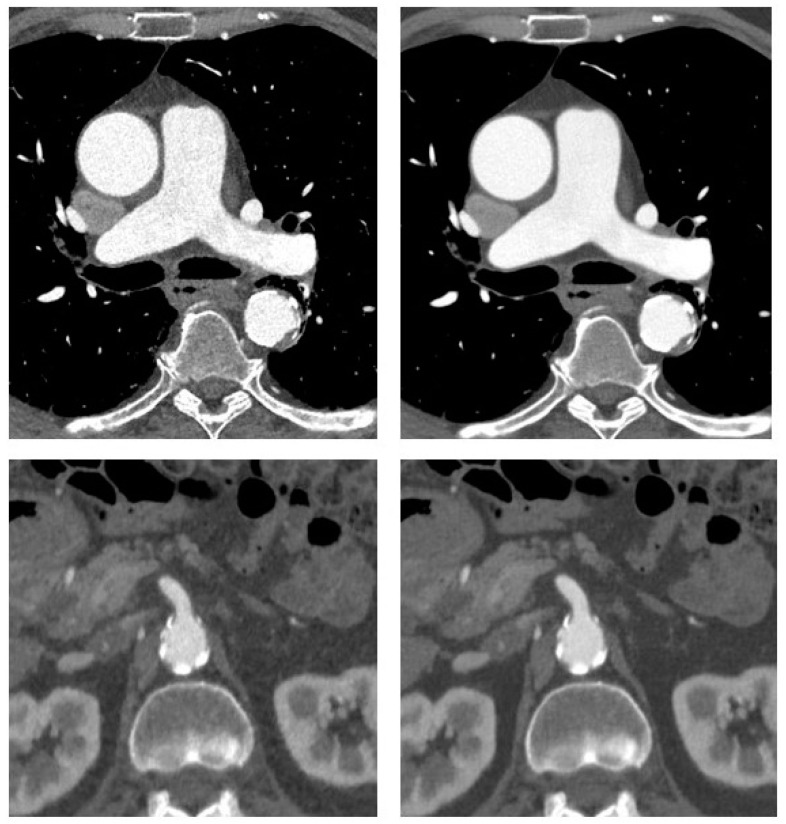
CT angiography of the aorta in a 72-year-old man with coronary artery disease and suspected aneurysm of the ascending aorta. Transverse sections at the level of the ascending aorta and superior mesenteric artery origin are shown with adaptive statistical iterative reconstruction V (ASIR-V 60%, **left**) and deep-learning-based reconstruction (DLIR-H, **right**). There is mild aneurysmatic dilatation of the ascending aorta with a maximum diameter of 4.3 cm. Some atherosclerotic plaque along the descending aorta is also noted.

Examples of the image reconstructions are shown in Figure 1 and Figure 2. 

### 2.4. Radiation Metrics and Image Reconstruction

The volume CT dose indices (CTDI_vol_) as well as the dose length products (DLP) were retrieved from the dose report stored in the picture archiving and communication system (IMPAX 6.5.3, Agfa HealthCare). 

### 2.5. Analysis of Objective Image Quality and Dose Efficiency

Quantitative analysis of image quality was performed by a radiologist (6 years’ professional experience) to determine image noise, the signal-to-noise ratio (SNR) and the contrast-to-noise ratio (CNR). For each patient and for each of the two reconstruction methods, a circle region of interest (ROI) was placed on an axial slice in the lumen of the ascending aorta (at the level of the main pulmonary artery), the descending thoracic aorta (at the level of the aortic valve), the abdominal aorta (at the level of the superior mesenteric artery origin), in the right iliac artery (at the level of the inguinal ligament) and in the paraspinal muscles. The paraspinal muscle was used as a reference because it provides homogeneous attenuation, and fat deposits were avoided. Image noise was defined as the standard deviation of the CT attenuation in the named intravascular locations. For each location, SNR was calculated as mean intravascular CT attenuation/intravascular image noise. CNR was calculated as (mean intravascular CT attenuation–CT attenuation in the paraspinal muscle)/image noise. 

### 2.6. Subjective Assessment of Diagnostic Confidence and Motion Artifacts

The analysis of image quality was performed independently by two radiologists in random order and blinded to each other’s evaluation results. Both observers had access to all axial source images, coronal and sagittal reformation images and were allowed to freely adjust window settings.

The subjective overall image quality was scored on a 5-point scale as follows for the respective localizations: 5 = excellent, optimal enhancement to allow clear assessment; 4 = good, good contrast of the aorta with minimal artifacts; 3 = sufficient, some artifacts, which still allows adequate assessment of the aorta; 2 = poor, inadequate image quality because of no contrast or too many artifacts; 1 = non-diagnostic. 

### 2.7. Statistical Analyses

Statistical analysis was performed with GraphPad Prism version 9.0.0 (GraphPad Software LLC) and SPSS version 27.0.0 (IBM). We tested for normal distribution using the Shapiro–Wilk test. Most but not all parameters of objective image quality were normally distributed and most but not all parameters of subjective image quality were not normally distributed. For simplicity, we decided to use non-parametric tests for all comparisons. Thus, all values were presented as the median and interquartile range (25 to 75 percentiles). The Wilcoxon signed rank test for matched pairs was used to compare image quality parameters between ASIR-V and DLIR. A two-tailed *p*-value was used. Subgroup analysis was performed to assess the performance of DLIR in obese patients (BMI ≥ 30 kg/m^2^) and normal-weight patients (BMI < 25 kg/m^2^). Weighted kappa was computed to assess interobserver agreement with a linear weighting scale. To account for multiple testing at four different anatomical levels of the aortoiliac vasculature, an adjusted alpha level of *p* < 0.01 was considered to indicate statistical significance. 

## 3. Results

### 3.1. Patient Characteristics

Patient characteristics are summarized in Table 2. We analyzed 51 consecutive patients (32 men, 19 women) with a median age of 69 years (interquartile range 57–74 years) who underwent CT angiography of the aorta on the same 256-detector-row CT. The median weight was 89 kg (interquartile range 72–100 kg) with a median BMI of 27.4 kg/m^2^ (interquartile range 23.9–31.0 kg/m^2^). A total of 17 patients in our cohort were obese with a BMI of ≥30 kg/m^2^, of which 11 were men and 6 were women.

### 3.2. Spectrum of Indications

Six main indications for the aortic CTA were defined: Acute aortic syndrome (*n =* 9), trauma (*n =* 2), aneurysm follow-up (*n =* 9), dissection (*n =* 3), preoperative (*n =* 9) and postoperative imaging (*n* = 19). The pre-operative examinations were performed to exclude relevant aortic aneurysms in patients planned for coronary bypass grafts (*n* = 5) or valvular heart surgery (*n* = 4). Postoperative/postprocedural examinations included 12 patients after open aortic repair (replacement of the ascending aorta ± aortic arch), 3 patients after thoracic endovascular aortic repair and 4 patients after hybrid procedures combining open replacement of the ascending aorta ± aortic arch with the implantation of a stentgraft into the descending aorta. Summarizing the main indications, 40 patients were examined in clinical routine as planned imaging while only 11 CTs were performed as emergency examinations. 

### 3.3. Radiation Dose

The median DLP for the ECG-gated thoracic acquisition was 126 mGy*cm (interquartile range 125–153 mGy*cm) and the median DLP for the abdominal, non-gated acquisition was 265 mGy*cm (interquartile range 246–298 mGy*cm). The median total DLP was 389 mGy*cm (range 344–418 mGy*cm).

### 3.4. Objective Image Quality

**Figure 3:**
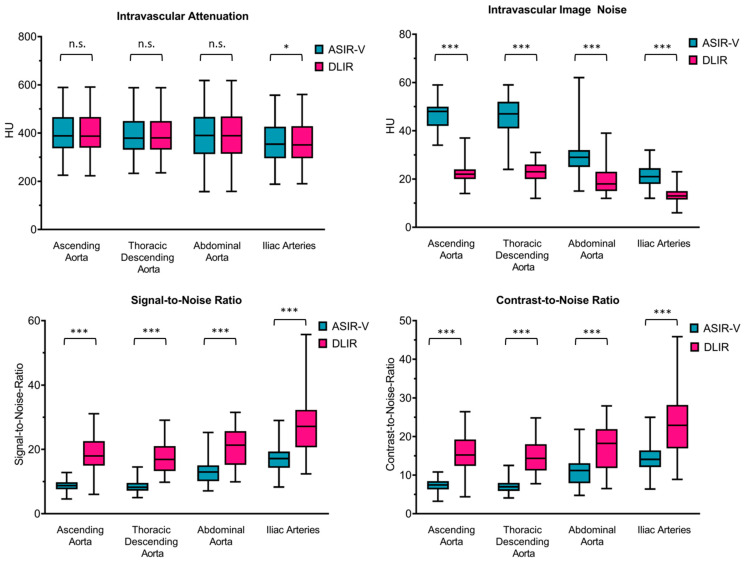
Objective image quality. Figure 3 data are shown as boxplots with the whiskers ranging from the lowest to the highest value, with the box extending from the 25th to the 75th percentile and the median plotted as the line inside the box. Turquoise: ASIR-V = adaptive statistical iterative reconstruction V, magenta red: DLIR = deep learning-based image reconstruction, HU = Hounsfield units. For comparisons between DLIR and ASIR-V, the level of statistical significance is shown as n.s. for not significant, * for *p* < 0.01 and *** for *p* < 0.0001.

The median attenuation values in the paraspinal muscle and vascular lumen were without relevant differences between ASIR-V and DLIR (see Figure 3). Compared to ASIR-V, DLIR reduced median image noise by slightly more than 50% for the ascending aorta (22 vs. 48 Hounsfield units (HU)) and the descending thoracic aorta (23 vs. 47 HU, both *p* < 0.0001). Correspondingly, median SNR roughly doubled for the ascending aorta (18 vs. 9) and the descending thoracic aorta (17 vs. 8, both *p* < 0.0001). The same effect size was seen for CNR in the ascending aorta (15 vs. 7) and the descending thoracic aorta (14 vs. 7, both *p* < 0.0001). There was a roughly 40% reduction in image noise for the abdominal aorta (18 vs. 29 HU) and the iliac arteries (13 vs. 21 HU, both *p* < 0.0001), with an equivalent improvement in SNR (21 vs. 13 for the abdominal aorta, 27 vs. 17 for the iliac arteries, both *p* < 0.0001) and CNR (18 vs. 11 for the abdominal aorta, 23 vs. 14 for the iliac arteries, both *p* < 0.0001). 

In obese patients (*n* = 17, median BMI 32.7 kg/m^2^), improvements in CNR ranged from 52% for the iliac arteries to 103% for the ascending aorta. In normal-weight patients (*n* = 17, median BMI 21.9 kg/m^2^), improvements in CNR were even more marked, ranging from 63% for the iliac arteries to 123% for the ascending aorta (Table 3).

All four patients after a hybrid procedure and all three patients after thoracic endovascular aortic repair had aortic endografts with metal struts in their thoracic descending aorta. In this group of seven patients, the median contrast-to-noise-ratio at the level of the stentgraft was 7 for ASIR-V and 13 for DLIR. In all other patients, the median contrast-to-noise-ratio in the descending thoracic aorta was 7 for ASIR-V and 15 for DLIR. Thus, the contrast-to-noise ratio in the DLIR reconstruction was slightly reduced in the presence of stentgrafts with metallic struts.

### 3.5. Subjective Image Quality

There was moderate to excellent interobserver agreement with respect to the subjective image quality, with weighted kappa values ranging from 0.456 to 0.822 (Table 4). The results of the visual evaluation of the subjective image quality are summarized in Table 5. Subjective image quality was at least “sufficient” (score 3) in all patients. No examinations received scores of 2 (“poor”) or 1 (“non-diagnostic “). For both readers, the median subjective image quality was superior for DLIR compared to ASIR-V at all anatomical levels (all *p* < 0.0001). The median subjective image quality was good for ASIR-V and excellent for DLIR at all anatomical levels (except for the iliac arteries for reader 1, rated as excellent in both ASIR-V and DLIR reconstructions).

## 4. Discussion

We observed that DLIR significantly reduces image noise and provides significantly higher SNR and CNR in CT angiography of the aorta than a state-of-the-art iterative reconstruction algorithm (ASIR-V). Subjective image quality was also significantly improved with DLIR compared to ASIR-V.

DLIR is a very recent technological innovation with only a small number of previous studies published in the literature. Initial phantom studies [13,14] demonstrated that image noise is lower with better high-contrast spatial resolution and task-based detectability in DLIR images compared to filtered back projection, hybrid iterative reconstruction and MBIR.

In a retrospective clinical study, Brady and colleagues evaluated the effect of DLIR on image quality for pediatric CT [15]. They compared DLIR with filtered back projection (FBP), statistical-based iterative reconstruction and MBIR. In their study, DLIR improved object detectability and reduced image noise compared to all other reconstruction algorithms, and radiologists consistently preferred DLIR images. Akagi and colleagues investigated DLIR in specific scenarios where excessive image noise is problematic. They found that DLIR can be used to preserve or even enhance image quality in abdominal CT for obese patients [16] and for ultra-high-resolution abdominal CT [6,17]. 

Little research has been published on DLIR in cardiovascular CT imaging. One study by Tatsugami and colleagues investigated the effects of DLIR on coronary CT angiography [3]. They observed that DLIR reduced image noise and improved objective and subjective image quality compared to a hybrid iterative reconstruction algorithm. The results are thus remarkably similar to our study in CT angiography of the aorta, although a DLIR algorithm developed by a different vendor was investigated. 

The DLIR algorithm investigated in our study allows the user to choose between low, medium and high strength. This setting determines how aggressively the image noise is modeled. Another recent study on coronary CT angiography included a comparison of medium- and high-strength DLIR images [18]. They found that DLIR-H (high strength) provided superior image quality compared to both DLIR-M (medium strength) and ASIR-V reconstructions with no differences in diagnostic accuracy [18]. In our study, we chose to investigate only the high-strength setting since our experience suggests that it provides the best image quality for CT angiography.

Since the DLIR-H reconstruction models the image noise most aggressively, there could be concerns whether such reconstruction could result in distortion of images with the potential risk of missing or misdiagnosing lesions. In our practice, we therefore routinely view both the conventional iterative reconstructions and the deep-learning based reconstructions. So far, we have not observed any cases in either this study cohort or in our clinical routine where the DLIR reconstruction removed or obscured pathological findings. Nevertheless, it should be evaluated in further studies whether this is a possible risk. Repeated CTA scans are often necessary for surveillance, post-surgical or post-interventional follow-up of aortic diseases [19]. Therefore, limiting radiation exposure is particularly important in CTA of the aorta. Our median CTDI_vol_ values (5.5 mGy for the ECG-gated acquisition of the chest and 6.4 mGy for the non-gated acquisition of the abdomen) are substantially below the German national diagnostic reference value for a CT angiography of the complete aorta (9.0 mGy, defined as the 75th percentile of a reference database) and even below the 25th percentile (7.0 mGy) [20]. This reflects the use of a high-end CT system and a dose-efficient protocol setup with reduced tube voltage (100 kV), prospective ECG-triggering and attenuation-based tube voltage modulation. On the other hand, image quality was rated as excellent in DLIR images for most patients—indicating that DLIR provides an opportunity for additional reductions in radiation dose. In our study, DLIR resulted in a noise reduction of 40–50% compared to ASIR-V. Image noise is inversely proportional to the square root of the CTDI_vol_ [21,22]. Thus, we project that, theoretically, the radiation exposure could be further reduced up to 60–75% with DLIR compared to ASIR with constant image noise. Further studies are needed to determine whether diagnostic confidence and accuracy are indeed maintained at such low radiation doses.

Due to the technical development of CT scanners, smaller volumes of the contrast agent are required for CT angiography of the aorta [23]. In our protocol, a fixed volume of 80 mL of the contrast agent with an iodine concentration of 400 mg/mL was used. Other studies have used weight-adjusted contrast volumes such as 1 mL per kg body weight or volumes adjusted for both bodyweight and tube potential selection [24,25,26]. The increase in the contrast-to-noise ratio achieved with DLIR could also be used to decrease the contrast volume instead of decreasing the radiation exposure (or to moderately decrease both). This choice will depend on the clinical scenario. In elderly patients with renal impairment, it would be prudent to invest the increase in CNR in decreasing the contrast volume rather than the radiation exposure.

In our CT-scans, we used the thinnest slice thickness (0.625 mm) to increase the spatial resolution in the *z*-axis. Thin slices are extremely relevant for correct diagnosis in aortic CTA as they allow for high-quality multiplanar reformats and 3D reconstructions. However, image noise increases with a decreasing slice thickness. Marco et al. have also shown that when using the ASIR-V algorithm, the effect on noise was dependent on slice thickness and different reconstruction kernels [27]. They observed a greater noise reduction at 2.5 mm than at 0.625 mm, especially for the soft-tissue kernel. In our study, we were able to show that with the DLIR algorithm, the noise is significantly below the values of ASIR-V even in thin slices, resulting in excellent image quality. 

Our study has a few limitations. The study population of 51 patients was relatively small although statistical power was more than sufficient considering the large observed differences in image quality and the paired sample design. Our analyses were retrospective and were conducted at a single institution. An analysis of diagnostic accuracy was not possible due to the lack of an external reference standard. The results were obtained with a single-vendor DLIR algorithm and are likely not transferable to the DLIR algorithms of other vendors. We did not compare the filtered back projection or MBIR, since our goal was to compare DLIR with an advanced iterative reconstruction algorithm that represents a clinical state-of-the-art method. Subjective image quality was rated by two radiologists, but measurements of objective image quality parameters were performed by a single observer. Therefore, inter-rater variability could only be analyzed for subjective image quality.

## 5. Conclusions

In conclusion, DLIR substantially improves objective and subjective image quality in CT angiography of the aorta beyond what can be achieved by state-of-the-art iterative reconstruction. The predicted potential for an additional reduction in radiation dose could be in the order of 60–75%. Whether this is truly feasible needs confirmation in further studies. 

## Figures and Tables

**Table 1:** CT Protocol.

Parameter	Value	
**Regions covered**		
Thoracic and abdominal aorta	43 patients	
Thoracic aorta only	8 patients	
**Acquisition parameters**		
Tube voltage	100 kV	
Tube current	tube current modulation	
Reference noise index	25	
ECG-triggering	75% of RR-interval (chest only)	
**Contrast protocol**		
Contrast volume	80 mL	
Contrast concentration	400 mg/mL	
Flow rate	4 mL/s	
Saline chaser	40 mL with 4 mL/s	
**Reconstruction parameters**		
Reconstruction method	ASIR-V 60%	DLIR-H
Reconstruction kernel	HD Stnd	HD Stnd/Stnd
Slice thickness	0.625 mm	0.625 mm
Slice increment	0.625 mm	0.625 mm
**Radiation metrics**		
CTDI_vol_ chest	5.5 (5.3–6.8) mGy	
CTDI_vol_ abdomen	6.4 (6.3–6.4) mGy	
DLP chest	126 (125–153) mGy*cm	
DLP abdomen	265 (246–298) mGy*cm	
Total DLP	389 (344–418) mGy*cm	

Table 1 data are shown as the median (25–75 percentile) for DLP and CTDI_vol_. ASIR-V = adaptive statistical iterative reconstruction V, DLIR-H = deep-learning-based reconstruction–high strength, CTDI_vol_ = volume computed tomography dose index, DLP = dose length product, HD = high definition, Stnd = standard.

**Table 2:** Patient characteristics.

	All Patients	Men	Women
No. of patients	51	32	19
Age (years)	69 (57–74)	64 (54–72)	70 (65–79)
Body weight (kg)	89 (72–100)	94 (84–106)	73 (61–81)
BMI (kg/m^2^)	27.4 (23.9–31.0)	27.8 (24.6–31.0)	26.1 (21.7–30.9)
Main indication for CTA			
Aneurysm follow-up	9	7	2
Dissection	3	1	2
preoperative	9	7	2
postop (incl. TEVAR)	19	10	9
Acute aortic syndrome	9	5	4
Trauma	2	2	0

Table 2 data are shown as median (25–75 percentile) for age, body weight and BMI. BMI = body mass index. TEVAR = thoracic endovascular aortic repair.

**Table 3:** Subgroup analysis.

	Obese Patients (*n =* 17)			Normal-Weight Patients (*n =* 17)		
Contrast-to-Noise-Ratio	ASIR-V	DLIR	*p*-Value	ASIR-V	DLIR	*p*-Value
Ascending Aorta	7 (6–8)	14 (12–16)	<0.0001	8 (8–9)	19 (15–22)	<0.0001
Thoracic descending aorta	7 (6–7)	13 (11–15)	<0.0001	8 (7–9)	18 (13–19)	<0.0001
Abdominal aorta	12 (8–13)	19 (15–24)	<0.0001	9 (7–12)	16 (11–21)	<0.0001
Iliac arteries	14 (12–15)	21 (19–25)	<0.0001	15 (13–16)	24 (19–28)	0.001

Table 3 data are shown as the median (25–75 percentile). ASIR-V = adaptive statistical iterative reconstruction V, DLIR = deep learning-based image reconstruction.

**Table 4:** Interobserver agreement of subjective image quality.

	ASIR-V	DLIR
Ascending aorta	0.659	0.509
Thoracic descending aorta	0.630	0.807
Abdominal aorta	0.552	0.456
Iliac arteries	0.706	0.822

Table 4 weighted kappa values are shown for interobserver agreement of subjective imaging quality ratings. ASIR-V = adaptive statistical iterative reconstruction V, DLIR = deep learning-based image reconstruction.

**Table 5:** Subjective image quality.

	ASIR-V	DLIR	*p*-Value
Reader 1			
Ascending aorta	4 (4–5)	5 (5–5)	<0.0001
Thoracic descending aorta	4 (4–5)	5 (5–5)	<0.0001
Abdominal aorta	4 (4–4)	5 (4–5)	<0.0001
Iliac arteries	5 (4–5)	5 (5–5)	<0.0001
Reader 2			
Ascending Aorta	4 (4–4)	5 (4–5)	<0.0001
Thoracic descending aorta	4 (4–4)	5 (5–5)	<0.0001
Abdominal aorta	4 (3–4)	5 (4–5)	<0.0001
Iliac arteries	4 (4–5)	5 (5–5)	<0.0001

Table 5 subjective image quality was scored on a 5-point scale (5 = excellent, 4 = good, 3 = sufficient, 2 = poor, 1 = non-diagnostic). Data are shown as the median (25–75 percentile). ASIR-V = adaptive statistical iterative reconstruction V, DLIR = deep learning-based image reconstruction.

## Data Availability

All supporting data is available from the correspondence author upon reasonable request.
